# Cross-Modality Whole-Heart MRI Reconstruction with Deep Motion Correction and Super-Resolution

**DOI:** 10.3390/s26051565

**Published:** 2026-03-02

**Authors:** Jinwei Dong, Wenhao Ke, Wangbin Ding, Liqin Huang, Mingjing Yang

**Affiliations:** 1 College of Physics and Information Engineering, Fuzhou University, Fuzhou 350116, China; 211110003@fzu.edu.cn (J.D.); 241120109@fzu.edu.cn (W.K.); hlq@fzu.edu.cn (L.H.); 2 School of Medical Imaging, Fujian Medical University, Fuzhou 350122, China; dingwangbin@fjmu.edu.cn

**Keywords:** cross-knowledge, MRI label, super resolution, motion correction, reconstruction, whole heart segmentation

## Abstract

Magnetic resonance imaging (MRI) inherently suffers from motion artifacts and inter-slice misalignment, primarily due to sequential slice acquisition and the prolonged scanning time required for dynamic cardiac motion. These acquisition-induced inconsistencies often lead to anatomically implausible representations of cardiac structures, impairing subsequent clinical analyses such as 3D reconstruction and regional functional assessment. On the other hand, acquiring high-resolution MRI demands extended scan durations that increase patient burden and potential health risks. To address this challenge, we propose a deep motion correction and super-resolution whole-heart reconstruction (DeepWHR) framework. It learns cardiac structure prior knowledge from computed tomography (CT) data, and transfers it to reconstruct cardiac structure from conventional misaligned and large slice thickness MRI images. Specifically, DeepWHR utilizes CT anatomy data to train a deep motion correction model that enables the network to capture structurally coherent and anatomically consistent representations, while MRI Finetune preserves modality-specific spatial characteristics, ensuring that the reconstructed results retain the intrinsic MRI data distribution. Furthermore, DeepWHR introduced an implicit neural representation module, which models continuous spatial fields, enabling multi-scale super-resolution structure reconstruction. Experiments on the CARE2024 WHS dataset validate that our method not only restores the spatial coherence of MRI-derived anatomical structures but also generates high-fidelity label representations suitable for downstream cardiac applications. This study demonstrates that DeepWHR transforms sparse, misaligned 2D label stacks into anatomically coherent, high-resolution 3D models, enhancing their reliability for clinical applications.

## 1. Introduction

**Figure 1:**
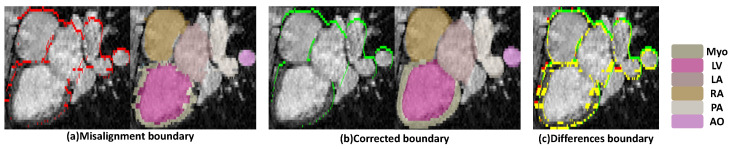
Impact of respiratory motion on MRI, causing inter-slice misalignment and structural deformation. (**a**) Shows the misaligned anatomical structures, (**b**) displays the corrected, anatomically accurate structures, and (**c**) highlights the differences between them, with boundary colors: red for misaligned boundaries, green for correct boundaries, and yellow for differences.

Accurate delineation of cardiac anatomy from magnetic resonance imaging (MRI) is fundamental for the quantitative evaluation and clinical interpretation of cardiovascular diseases [1]. Despite MRI being a non-invasive modality renowned for its rich soft tissue contrast and serving as the gold standard for left ventricular assessment [2,3], its acquisition characteristics frequently introduce motion-related artifacts and spatial inconsistencies. Specifically, cardiac and respiratory motion occurring across multiple breath holds often manifests as inter-slice misalignment [4,5] (see Figure 1), yielding anatomically implausible representations. Compounding this, the limited through-plane resolution of routine cardiac MRI results in sparse longitudinal sampling. These imperfections are particularly detrimental to advanced downstream tasks beyond simple volume calculation. However, the generation of high-fidelity 3D whole-heart models is critical for the advancement of computational medicine, serving as the geometric foundation for computational fluid dynamics (CFD) simulations, electrophysiological modeling, and virtual surgical planning. In these contexts, geometric continuity is paramount; even minor stair step artifacts caused by inter-slice misalignment can result in nonmanifold meshes, introducing artificial turbulence or conduction blocks that compromise diagnostic reliability. To mitigate these geometric errors, motion correction is typically employed to spatially realign the disparate 2D slices into a geometrically consistent 3D volume [6,7,8]. However, conventional motion correction relying on intensity-based registration is often suboptimal. These methods are frequently misled by MRI-specific artifacts, such as bias fields and flow voids, and strive to align pixel intensities without guaranteeing the preservation of anatomical integrity.

Recognizing these constraints, recent research has increasingly shifted towards label-domain processing [9,10], which aims to decouple geometric correction from erratic signal variations by focusing directly on anatomical boundaries. Furthermore, given the low through-plane resolution inherent to cardiac MRI, it is widely recognized that motion correction alone is insufficient; super-resolution (SR) is an indispensable step to recover missing longitudinal information and eliminate residual stair step artifacts.

While conventional segmentation networks struggle to reconstruct high-fidelity volumes from motion-corrupted inputs, various methodologies have been proposed to bridge this gap. Prevalent strategies typically employ spatial alignment via intensity-based registration [11] or non-rigid deformation fields; however, these methods frequently fail when image features are ambiguous or artifacts are severe. Diverging from intensity-driven paradigms, subsequent approaches have utilized auto-encoders to enforce shape constraints during joint segmentation and super-resolution [12,13]. Yet, these deep learning methods remain fundamentally limited by the quality of the training data. When the available ground truth itself harbors motion artifacts, a common occurrence in the absence of laborious manual correction, the network inevitably internalizes a flawed shape prior. Similarly, recent end-to-end frameworks [14] trained on synthetic perturbations of inherently misaligned data often lack a valid anatomical basis, exacerbating structural distortions when correcting significant misalignments.

Overcoming the bottleneck of generating biologically plausible 3D structures from corrupted input requires looking beyond the MRI modality. Obtaining perfectly aligned, high-resolution MRI ground truth is notoriously difficult [15,16,17,18,19]; in contrast, computed tomography (CT) naturally offers dense, isotropic spatial resolution with minimal motion artifacts. Rather than acting merely as a data substitute, CT serves as an ideal source for learning robust volumetric anatomical priors. By leveraging the geometric integrity inherent to CT, we can extract shape manifolds that are independent of modality-specific intensity variations, thereby providing a strong constraint for correcting the geometric degradation found in MRI acquisitions.

In this work, we propose a two-stage supervised framework designed to reconstruct motion-corrected and SR-label representations without relying on paired high-quality MRI data. The core of our methodology is a cross-modality structural knowledge transfer strategy. We employ a latent code-based shape prior model, pretrained on high-fidelity CT data, to implicitly rectify structural inconsistencies by projecting MRI observations onto a learned anatomical manifold. Subsequently, by incorporating Coordinate-based Implicit Neural Representation (INR), the framework models the label field as a continuous 3D function, effectively bridging inter-slice discontinuities to support arbitrary-scale SR.

The main contributions of this work are summarized as follows:

- We propose a label-domain refinement framework that shifts the paradigm of motion correction from intensity-based alignment to structural restoration. By decoupling geometric consistency from the complex signal variations in MRI, our approach directly repairs inter-slice discontinuities based on anatomical topology, enabling robust 3D reconstruction even when the underlying image data are ambiguous.
- We introduce a cross-modality optimization strategy that leverages CT-derived latent shape priors for explicit motion correction, coupled with INR to achieve continuous, high-fidelity super-resolution.
- Validated on the CARE2025 WHS dataset, our method demonstrates superior spatial coherence and surface quality, accurately delineating fine substructures while maintaining computational efficiency.

## 2. Related Work

### 2.1. Motion Correction

Early attempts to address inter-slice misalignment primarily relied on post-processing techniques centered on geometric registration. Traditional methods, such as polynomial fitting [20] and rigid registration [21], attempt to realign slice stacks by maximizing intensity correlation or boundary overlap between adjacent frames. While effective for global respiratory shifts, these methods often struggle with non-rigid local deformations and lack semantic awareness, frequently failing when image contrast is low or artifacts are severe.

With the advent of deep learning, data-driven approaches have improved automation. Dangi et al. [22] proposed a slice-wise regression network to localize the left ventricular center, aligning slices based on predicted landmarks. Similarly, Chang et al. [23] utilized recurrent neural networks (RNNs) to enforce temporal consistency across the slice stack. However, these methods remain fundamentally rooted in 2D spatial cues. They optimize for relative displacement between consecutive slices rather than holistic 3D integrity, often resulting in volumes that are locally smoothed but topologically distorted in the through-plane direction.

To overcome the limitations of purely geometric alignment, recent research has investigated the use of anatomical shape priors. Auto-encoder architectures have been widely employed to learn compact representations of cardiac shapes, serving as regularization terms during segmentation [12,24]. However, a critical bottleneck in MRI-based shape learning is the quality of the training data. Since ground-truth 3D MRI labels often contain motion artifacts or rely on interpolation, networks trained exclusively on MRI data risk internalizing flawed shape distributions.

While cross-modality domain adaptation has been explored for image synthesis or segmentation transfer, its potential for explicit structural correction remains underutilized. Existing domain adaptation methods typically focus on intensity style transfer to bridge the domain gap. In contrast, our proposed deep structure-consistency learning (DSL) framework focuses on transferring the structural manifold. By leveraging the intrinsic isotropy and completeness of CT data, we aim to learn a robust, modality-invariant topological prior. This approach differs from previous methods by treating motion correction not as a pixel-wise registration task, but as a manifold projection problem, where misaligned MRI predictions are rectified by mapping them onto the valid latent space defined by CT anatomy.

### 2.2. Z-Axis High-Resolution Volume Generation

The reconstruction of isotropic 3D volumes from anisotropic cardiac MRI has traditionally relied on interpolation techniques or single-image super-resolution (SISR) algorithms adapted for volumetric data. Early approaches employed B-spline or linear interpolation to upsample the through-plane dimension, often resulting in blurred edges and phantom artifacts. With the advancement of deep learning, more sophisticated frameworks have emerged. For instance, Wang et al. [3] introduced a latent optimization framework to perform joint motion correction and super-resolution. Similarly, other works have framed the problem as image inpainting [25] or slice interpolation.

However, a fundamental limitation of these intra-modality approaches is their exclusive reliance on the MRI data itself. When the input slices are corrupted by severe respiratory motion or exhibit large anatomical gaps, the network lacks a reliable ground truth to guide the reconstruction. Consequently, these methods often inadvertently amplify intrinsic artifacts, yielding 3D volumes that are higher in resolution but geometrically deformed or structurally incoherent (hallucinated structures).

To address the sparsity of MRI data, complementary research has focused on geometric shape completion. Techniques involving point cloud completion [26] or mesh deformation [27] attempt to infer missing geometries based on learned surface smoothness. While these methods enhance continuity, they typically depend on surface smoothing algorithms that can obscure fine grained morphological details.

Our work diverges from these isolated reconstruction tasks by introducing a cross-modality structural prior. We posit that valid cardiac anatomy resides within a predictable manifold that is best learned from high-fidelity CT data, rather than sparse MRI. Unlike previous methods that perform pixel-level extrapolation, we leverage the dense, isotropic structural scaffold provided by CT. By guiding the super-resolution process with a learned CT-derived shape manifold, we aim to hallucinate missing *z*-axis information that is anatomically plausible, effectively bridging the gap between anisotropic acquisition and high-fidelity 3D modeling.

## 3. Method

**Figure 2:**
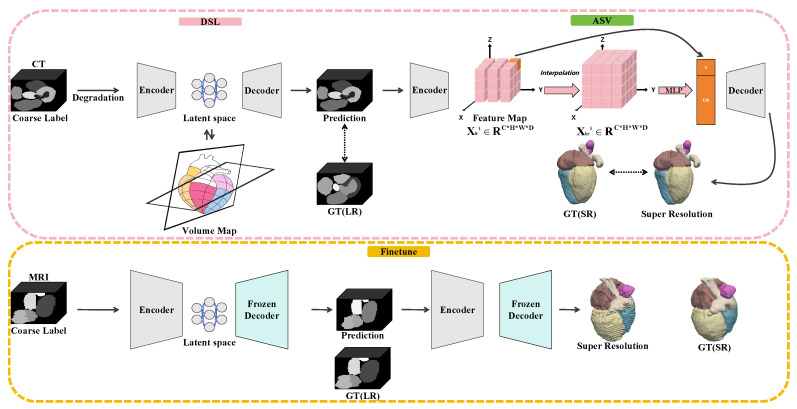
An illustration of the DeepWHR framework for cardiac MRI reconstruction. The pipeline consists of two cascaded components: deep structure-consistency learning (DSL) for motion correction and anisotropic *z*-axis super-resolution (ASV) for continuous volumetric generation.

The overall framework, illustrated in Figure 2, comprises two integrated stages: deep structure-consistency learning (DSL) and anisotropic *z*-axis super-resolution (ASV). The first stage, DSL, addresses inter-slice misalignment by aligning spatially displaced structures along the through-plane axis. Leveraging a cross-modality strategy, the network first learns robust shape priors and stable inter-slice relationships from CT pretraining. Subsequent finetuning of MRI data adapts these priors to modality-specific anatomical variations, ensuring structural correction without rigidly imposing CT morphology.

Following motion correction, the ASV stage further enhances volumetric resolution by modeling the label field as a continuous spatial function using an Implicit Neural Representation. This formulation effectively bridges residual gaps between slices, generating high-fidelity, smoothly varying 3D label volumes. Both stages are optimized using multi-objective losses that balance voxel-wise fidelity with volumetric overlap constraints, providing a unified solution for restoring spatial consistency and anatomical continuity suitable for reliable quantitative analysis. The following subsections detail the architecture and training strategies for (1) motion correction and (2) super-resolution.

### 3.1. Shape Prior Modeling via Latent Volume Mapping

We formulate motion correction as a supervised reconstruction problem governed by a learned anatomical manifold. The objective is to train a mapping function that projects a motion-corrupted volume $\tilde{X}$ onto its corresponding high-fidelity, motion-free ground truth $X_{gt}$. Since acquiring paired motion-corrupted and clean MRI data is clinically infeasible, we leverage high-resolution CT data to establish a robust shape prior, transferring this structural knowledge to the MRI domain via a latent volume representation.

#### 3.1.1. Data Degradation and Input Formulation

Let $D_{CT}={\left\{X_{CT}^{\left(i\right)}\right\}}_{i=1}^{N}$ denote a dataset of high-quality, isotropic CT label volumes. To force the network to learn invariance to motion artifacts, we introduce a stochastic degradation function T(·). During the pretraining phase, we generate synthetic training pairs by applying random slice-wise translations and anisotropic downsampling to the CT ground truth:

(1) $$\tilde{X}=T\left(X_{CT}\right),\;\tilde{X}\in R^{C\times D\times H\times W}$$

Here, $\tilde{X}$ serves as the input to the network, mimicking the stair-step artifacts and resolution anisotropy typical of clinical CMR, while $X_{CT}$ serves as the reconstruction target.

#### 3.1.2. Latent Volume Map Construction

We employ a 3D Convolutional Autoencoder architecture to learn the anatomical manifold. The network consists of a probabilistic encoder $E_{\phi }$ and a decoder $D_{\theta }$, specifically designed to handle the anisotropic nature of cardiac image stacks.

The encoder $E_{\phi }$ transforms the degraded input volume $\tilde{X}\in R^{C_{in}\times D\times H\times W}$ into a feature representation through a sequence of convolutional blocks. A critical design choice is the implementation of anisotropic downsampling. While standard 3D networks typically employ isotropic pooling, we utilize Maxpooling operations with a kernel size and stride of (1, 2, 2). This strategy reduces the spatial resolution in the in-plane dimensions (H,W) by a factor of 4 to extract high-level semantic features while preserving the original resolution along the longitudinal axis (*D*).

The output of the encoder is defined as the Latent Volume Map, denoted as Z. Unlike global feature vectors used in classification tasks, Z is a 4D tensor that retains spatial structure:

(2) $$Z=E_{\phi }\left(\tilde{X}\right),\;Z\in R^{C_{latent}\times D\times \frac{H}{4}\times \frac{W}{4}}$$

where $C_{latent}$ (set to 64 in our implementation) represents the number of feature channels. This structure allows Z to function as a spatially preserved compressed representation. By maintaining the depth dimension *D*, the map encodes the slice-wise progression of cardiac anatomy, while the compressed H/4×W/4 grid forces the network to discard high-frequency local noise (such as jagged boundaries caused by motion) and retain only the dominant topological components of the heart chambers.

The decoder $D_{\theta }$ mirrors the encoder structure, employing anisotropic upsampling (scale factor (1, 2, 2)) to restore the original spatial resolution. It maps the features from the latent manifold Z back to the label space:

(3) $$\hat{X}=D_{\theta }\left(Z\right)\;$$

During training, the objective is to minimize the reconstruction error between $\hat{X}$ and the clean ground truth $X_{CT}$. This forces the Latent Volume Map Z to encode a noise-invariant representation of the 3D geometry, effectively learning to project the geometrically distorted input $\tilde{X}$ onto the nearest anatomically valid configuration defined by the CT priors.

#### 3.1.3. Cross-Modality Transfer: From CT to MRI

To adapt this CT-derived prior to the MRI domain, we implement a sequential transfer learning strategy:

- CT pretraining (manifold learning): The network is first fully trained on the synthetic pair set $\left\{{\tilde{X}}_{CT},X_{CT}\right\}$. In this phase, the Latent Volume Map Z captures the generic, modality independent topological invariants of cardiac anatomy, establishing a robust structural baseline.
- MRI finetune: Subsequently, the model is finetuned on a smaller set of MRI data with manually corrected labels. During this process, the encoder weights $E_{\phi }$ are updated to accommodate MRI-specific spatial characteristics, while the decoder $D_{\theta }$ is frozen to preserve the structural smoothness learned from CT. This ensures that the network retains its capability for shape rectification while accurately adapting to the specific morphological distribution of the MRI modality.

### 3.2. Implicit Neural Reconstruction

The super-resolution module enhances the through-plane resolution of cardiac-label volumes by reconstructing continuous anatomical structures along the *z*-axis. Conventional volumetric upsampling treats the depth dimension as discrete, often generating layer-wise discontinuities that compromise anatomical plausibility. To address this, an implicit neural representation is introduced to model the label distribution as a spatially continuous function. This representation naturally interpolates structural transitions between adjacent slices, allowing the model to reconstruct coherent and anatomically consistent 3D shapes from anisotropic or motion-corrected inputs.

Let $X^{\mathrm{lr}}\;\in \;{\left\{0,\;1\right\}}^{C\times D_{\mathrm{lr}}\times H\times W}$ be the low-resolution label tensor with *C* classes and depth $D_{\mathrm{lr}}$. The task is to predict a high-resolution label map $X^{\mathrm{hr}}\;\in \;R^{C\times D_{\mathrm{hr}}\times H\times W}$, where $D_{\mathrm{hr}}=s_{z}D_{\mathrm{lr}}$. A normalized coordinate set ${\left\{\left(x_{i},y_{i},z_{i}\right)\right\}}_{i=1}^{N}\;\subset \;{\left[-1,\;1\right]}^{3}$, $N=D_{\mathrm{hr}}HW$, indexes the target voxels. Trilinear sampling provides coarse semantic anchors:

(4) $${\hat{X}}_{:,i}=S\;\left(X^{\mathrm{lr}},\left(x_{i},y_{i},z_{i}\right)\right)\in R^{C},\;$$

which are combined with spatial coordinates and embedded by a coordinate conditioned multilayer perceptron $\phi :R^{C+3}\;\to \;R^{C^{\prime }}$ to form continuous features:

(5) $$F_{i}=\phi \left(\left[x_{i},y_{i},z_{i},{\hat{X}}_{:,i}\right]\right)\in R^{C^{\prime }},\;F\in R^{C^{\prime }\times D_{\mathrm{hr}}\times H\times W}.\;$$

This implicit mapping enables smooth variation in structures along the depth dimension, capturing anatomical continuity that cannot be achieved by discrete convolutional kernels. A shallow encoder decoder operating at $D_{\mathrm{hr}}$ further integrates the implicit representation with an explicitly interpolated label tensor $X^{↑}$ through concatenation: $\left[M=\mathrm{concat}\left(X^{↑},F\right),\right]$ producing logits Z=ψ(M) and class probabilities P=softmax(Z). The explicit interpolation preserves the topology of the input labels, while the implicit branch enriches spatial continuity and local coherence.

Training minimizes a dual objective loss that constrains both voxel-wise fidelity and volumetric coherence:

(6) $$L_{\mathrm{SR}}=\lambda_{1}L_{L1}\left(P,Y\right)+\lambda_{2}L_{\mathrm{Dice}}\left(P,Y\right),\;\lambda_{1},\lambda_{2}>0.\;$$

The first term enforces intensity-level similarity between the predicted and reference labels using the voxel-wise $L_{1}$ distance:

(7) $$L_{L1}\left(P,Y\right)=\frac{1}{\left|\Omega \right|}\sum_{i\in \Omega }{∥P_{i}-Y_{i}∥}_{1},\;$$

and the second term measures volumetric overlap using the multi class Dice formulation:

(8) $$L_{\mathrm{Dice}}\left(P,Y\right)=1-\frac{2\sum_{c=1}^{C}\sum_{i\in \Omega }P_{c,i}Y_{c,i}}{\sum_{c=1}^{C}\left(\sum_{i\in \Omega }P_{c,i}^{2}+\sum_{i\in \Omega }Y_{c,i}^{2}\right)+\varepsilon }.$$

Here, Ω denotes the voxel set and ε a small stability constant. The $L_{1}$ term directly penalizes deviations from one-hot high-resolution targets and stabilizes optimization under class imbalance, while the Dice term enforces volumetric overlap and boundary integrity. Their combination ensures that the reconstructed labels remain numerically faithful and morphologically coherent, achieving both voxel-level accuracy and global anatomical consistency. CT labels, which exhibit structurally continuous volumes, are used for pretraining through a degradation reconstruction paradigm:

(9) $$\min_{\theta }\;E_{Y∼D_{\mathrm{CT}}}\;L_{\mathrm{SR}}\;\left(F_{\theta }\left({↓}_{z}Y\right),Y\right),\;$$

embedding stable depth continuity into parameters θ. Finetuning of MR data refines this prior without allowing CT characteristics to dominate:

(10) $$\min_{\theta }\;E_{\left(X,Y\right)∼D_{\mathrm{MR}}}\;L_{\mathrm{SR}}\;\left(F_{\theta }\left(X\right),Y\right).\;$$

Differential learning rates preserve the pretrained encoder’s structural understanding while adapting the decoder to MR-specific morphology. The modality-aware training ensures that the network restores through-plane resolution guided by CT-informed anatomical priors, yet preserves MR-specific features, preventing homogenized or template like label generation.

By treating the through-plane dimension as a continuous spatial function and supervising reconstruction with both voxel-level and volumetric criteria, this formulation achieves anatomically consistent super-resolution. It reconstructs cardiac structures with smooth cross-slice transitions, enhancing morphological realism while maintaining modality-specific integrity.

## 4. Results

### 4.1. Dataset and Preprocessing

The data used in this study are sourced from the CARE2024 Whole Heart Segmentation (WHS) Challenge [28,29,30]. To facilitate model training and evaluation, we split the WHS dataset into two subsets, each corresponding to a distinct cohort. The primary training cohort consists of 80 cases from a single clinical center, including 50 CT and 30 MRI volumes. Specifically, the 50 CT volumes served as the pretraining set to establish the shape prior manifold, while the MRI data were split into two subsets: 15 manually corrected volumes for modality-specific finetune, and 15 real clinical scans reserved as the real MRI test set for qualitative visual assessment. For manually corrected MRI, a cardiac radiologist with over five years of experience manually corrected the original MRI labels using multi-planar views to reduce motion artifacts and smooth the jagged boundaries of the anatomical walls, establishing the super-resolution ground truth (SR GT). To simulate clinical anisotropic acquisition, we downsampled the uncorrected original labels by removing even-numbered slices, creating motion-corrupted low-resolution (LR) inputs. Correspondingly, the SR GT was downsampled to generate LR GT targets.

Additionally, addressing the lack of isotropic ground truth in clinical MRI, we constructed a synthetic test set (Simulate MRI) for quantitative benchmarking. This set comprises 30 CT volumes from another clinical center, which underwent artificial degradation to simulate MRI-specific anisotropy and respiratory motion artifacts. Detailed volumetric statistics per chamber are shown in Table 1. This design enables rigorous evaluation using standard metrics including Dice Similarity Coefficient (Dice), 95% Hausdorff Distance (HD95), and Average Symmetric Surface Distance (ASSD) against high-fidelity ground truth.

For preprocessing, all volumes were cropped to a uniform size of 256×256×256 pixels centered on the cardiac region. During the CT pretraining phase, we employed a specialized augmentation strategy involving slice-specific rigid transformations to simulate respiratory misalignment and synthetic noise to mimic segmentation defects. This forces the network to learn robust anatomical rectification. Conversely, no artificial degradation was applied during the MRI finetuning stage, ensuring the model adapts the CT-derived priors to the intrinsic spatial distribution of real MRI anatomy without overfitting to synthetic artifacts.

### 4.2. Implementation Details

The framework was implemented in PyTorch 2.9.0 and trained on a workstation equipped with a single NVIDIA RTX 5090 GPU. The network parameters were optimized using the Adam optimizer with a learning rate of $1\times 10^{-4}$ for 1000 epochs. The loss function weights were set to $\lambda_{1}=\lambda_{2}=0.5$.

### 4.3. Motion Correction Study

#### 4.3.1. Visual Assessment on Clinical MRI

To evaluate the practical efficacy of our framework in a clinical setting, we conducted qualitative visual assessments on the Real MRI Test Set. This dataset consists of 15 clinical scans exhibiting varying degrees of respiratory motion artifacts, serving as a robust testbed for analyzing anatomical restoration in real-world scenarios.

**Figure 3:**
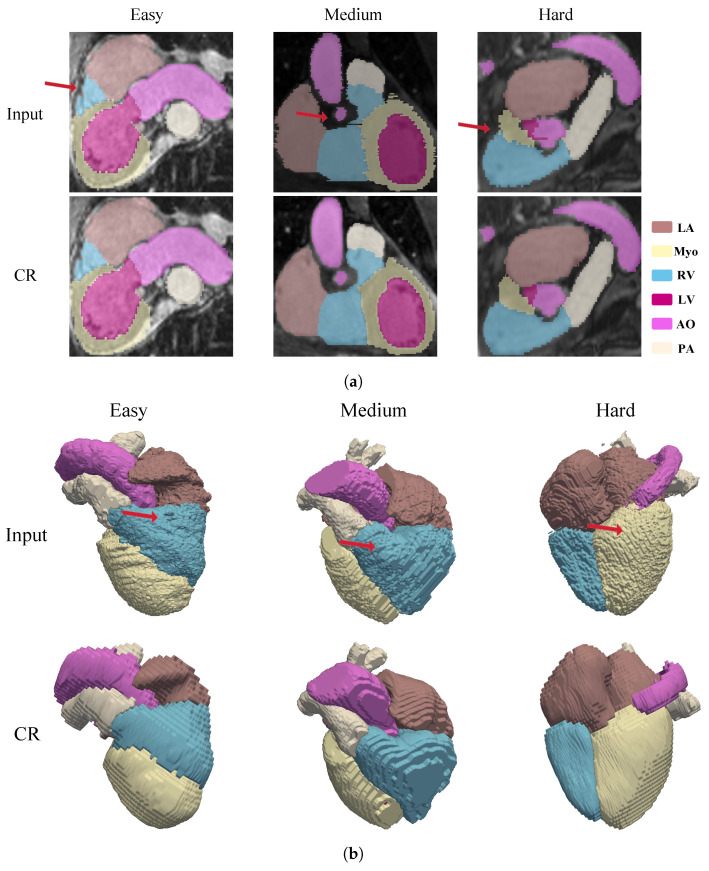
The view of motion corrected cardiac MRI labels in 2D and 3D views. The proposed framework effectively restores structural continuity and preserves anatomical plausibility across varying levels of respiratory induced misalignment. (**a**) The 2D visualization of motion correction results across easy, medium, and hard levels. Top: before correction; bottom: after correction (CR). Red arrows highlight regions with effective inter-slice alignment correction. (**b**) The 3D visualization of motion correction results for corresponding cases. Upper: original 3D reconstruction; lower: corrected results (CR). Red arrows indicate improved through-plane continuity.

As illustrated in Figure 3, the proposed method effectively corrects motion-induced inconsistencies across slices. The improvement is particularly evident in the 2D visualization (Figure 3a), where the characteristic jagged boundaries and inter-slice displacements are substantially smoothed.-specific attention is drawn to the right ventricular blood cavity (RV) and the myocardium of the left ventricle (Myo), structures that are typically most susceptible to severe distortions. Following correction, the stair-step artifacts along the ventricular walls are mitigated, and the pitted surface irregularities are replaced by coherent, continuous boundaries.

This structural recovery is further corroborated by the 3D volumetric rendering in Figure 3b. The corrected volumes display improved global alignment and topological consistency, effectively compensating for respiration-induced displacement. The results indicate that the network successfully preserves the intrinsic spatial characteristics of the original data while producing anatomically plausible reconstructions that conform to physiological expectations across different motion amplitudes.

#### 4.3.2. Quantitative Evaluation on Simulated MRI

To rigorously quantify the reconstruction performance, we utilized the synthetic test set, which provides ground-truth labels for benchmarking. We employed metrics including Dice, HD95 and ASSD to assess volumetric overlap and boundary fidelity.

Table 2 presents the comparative results against several established baselines. Traditional intensity-based registration, represented by Stolt et al., provides a fundamental baseline for rigid alignment (Dice 0.8620) but faces limitations in addressing complex non-rigid gaps due to the lack of semantic guidance. Similarly, the method by Sander et al. (RCH3), which incorporates CT priors, achieved a Dice of 0.8057 and a high HD95 of 47.62 mm, highlighting the difficulty of transferring cross-modality knowledge without an effective manifold projection mechanism. In the deep learning domain, the rigid correction strategy of Chen et al. demonstrated improved alignment (Dice 0.8659), while the non-rigid latent optimization approach of Wang et al. yielded a Dice of 0.8390 but suffered from a higher boundary error (HD95 45.50 mm).

In comparison, our method achieved the highest performance across all metrics, with a Dice score of 0.8778, an HD95 of 22.88 mm, and an ASSD of 1.34 mm. These improvements over the baselines indicate that learning shape consistency via a latent volume map yields better alignment than predicting slice-wise displacement or relying solely on intensity optimization. The significantly lower HD95 score further suggests that our approach effectively minimizes outliers and preserves boundary integrity during the reconstruction process.

**Figure 4:**
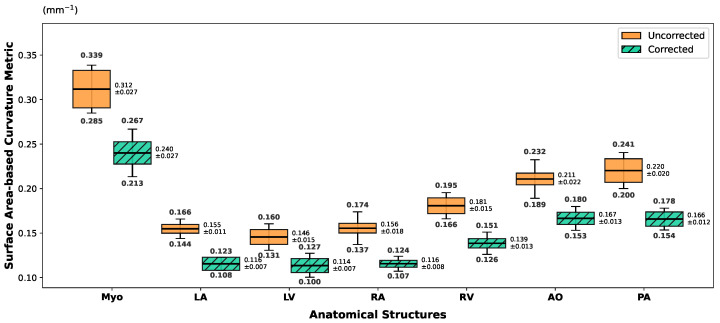
Visualization of the mean curvature intensity ($\bar{\kappa }$) (surface-to-volume ratio, unit: ${\mathrm{mm}}^{-1}$) across different tissue structures. The plots display the average $\bar{\kappa }$ values stratified by motion severity: orange represents hard (severe), red represents medium, and yellow represents easy cases. Solid lines denote the uncorrected baseline (${\bar{\kappa }}_{pre}$), while dashed lines indicate motion-corrected results (${\bar{\kappa }}_{post}$). The evident downward shift from solid to dashed lines, particularly in the hard and medium groups, demonstrates the effective removal of motion-induced stair-step artifacts, reflecting a reduction in surface area relative to volume and the restoration of smooth anatomical boundaries.

However, relying solely on volumetric overlap metrics offers an incomplete assessment of anatomical plausibility, particularly concerning surface continuity. High Dice scores can sometimes obscure the presence of unnatural stair-step artifacts characteristic of through-plane misalignment. To address this limitation and explicitly quantify the geometric smoothness, we introduce the Surface Curvature Variation Rate ($R_{\kappa }$) as a complementary descriptor, with the underlying curvature intensity profiles illustrated in Figure 4.

Surface curvature serves as a critical proxy for the three-dimensional bending degree of anatomical structures. In this study, we derive the curvature implicitly via the variation in the discrete surface area of the segmented labels. Let Ω represent the set of voxels belonging to a specific tissue label, and ∂Ω denote the boundary surface of this region. The total surface area *S* is calculated by aggregating the exposed faces of the boundary voxels:

(11) $$S\left(\Omega \right)=\sum_{v\in \partial \Omega }A\left(v\right)\;$$

where A(v) represents the discrete surface area contribution of voxel v. Based on the isoperimetric principle, for a region of fixed volume *V*, motion-induced jaggedness artificially inflates the surface area, indicating higher shape irregularity. We thus define the mean curvature intensity $\bar{\kappa }$ as the surface-to-volume ratio, formulated as $\bar{\kappa }=S\left(\Omega \right)/V\left(\Omega \right)$. To measure the correction effect, we calculate the relative curvature variation rate $R_{\kappa }$ between the uncorrected state (${\bar{\kappa }}_{pre}$) and the corrected state (${\bar{\kappa }}_{post}$):

(12) $$R_{\kappa }=\frac{|{\bar{\kappa }}_{post}-{\bar{\kappa }}_{pre}|}{{\bar{\kappa }}_{pre}}\;$$

This metric holds significant clinical value as an objective standard for assessing geometric restoration. Motion artifacts typically manifest as high-frequency boundary misalignments, resulting in abnormal curvature spikes. By quantifying the change in surface complexity, $R_{\kappa }$ effectively differentiates correction paradigms. While rigid correction methods often fail to resolve local deformations, effective non-rigid correction restores smooth physiological boundaries, reflected by a substantial reduction in geometric irregularity.

Analyzing organ-specific responses reveals distinct trends across motion severities. Under severe motion, substantial reductions in curvature intensity ($\bar{\kappa }$) occur in the ventricles and aorta, signifying the effective mitigation of large-scale surface fluctuations caused by misalignment. In medium motion settings, reductions are moderate and localized primarily to ventricular structures, indicating a shift toward refining local boundary consistency. Conversely, for easy motion, curvature remains stable across most tissues, confirming that the framework preserves originally coherent structures without introducing artificial smoothing or deformation. Collectively, these results validate $R_{\kappa }$ as a sensitive quantitative metric for evaluating structural restoration.

#### 4.3.3. Shape Distribution Shift Before and After Correction

**Figure 5:**
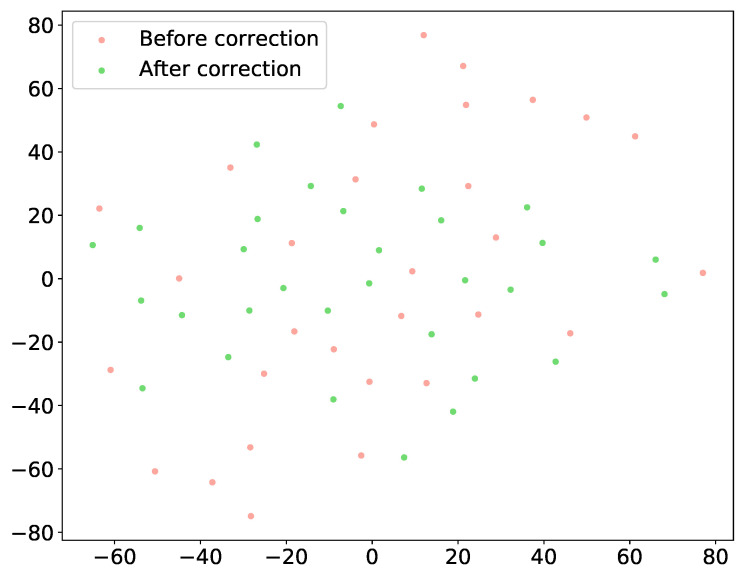
Shape distribution shift before and after correction on Simulated MRI datasets. Uncorrected predictions (red) are scattered and irregular due to motion artifacts; corrected ones (green) form a structured and compact manifold.

To verify that our model effectively projects motion-corrupted data onto a valid anatomical manifold without losing patient-specific details, we visualized the distribution of latent shape features using t-SNE. Figure 5 illustrates the feature space of the Simulated MRI dataset before and after correction. The distribution of the uncorrected predictions (red points) is highly scattered and irregular, reflecting the stochastic nature of motion artifacts and inter-slice misalignment. In contrast, the corrected representations (green points) are mapped onto a more structured and compact manifold. Importantly, the corrected points do not collapse to a single centroid but maintain relative distances, indicating that the network successfully removes geometric noise while preserving the unique anatomical variability inherent to each subject.

#### 4.3.4. Qualitative Evaluation of Real MRI

**Figure 6:**
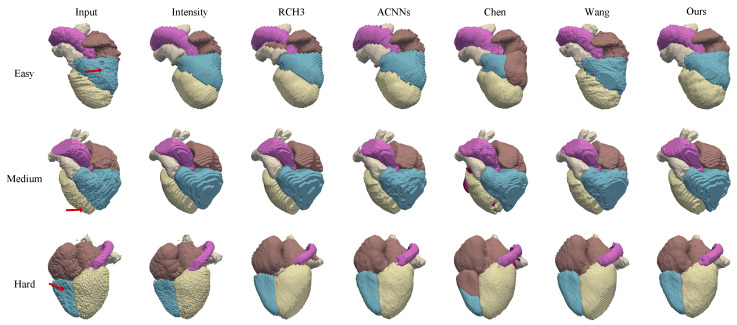
3D reconstruction visualization results from real clinical MRI. The figure compares our method with other methods. Red arrows highlight regions where DeepWHR produces more plausible surfaces compared to competing methods.

To evaluate the practical efficacy of our framework in a clinical setting, we conducted qualitative visual assessments on the real MRI test set. As illustrated in Figure 6, we compared the 3D reconstruction results of our method against five other approaches across cases with varying degrees of motion artifacts. The input column reveals severe stair-step artifacts and jagged anatomical boundaries caused by respiratory motion and sparse sampling, particularly evident in the right ventricle and left ventricle. While comparison methods such as intensity-based registration and RCH3 reduce some misalignment, they often struggle to maintain global shape consistency or fail to fully eliminate high-frequency geometric noise in the hard cases. In contrast, our method consistently generates smooth, biologically plausible surfaces with coherent topology. By effectively utilizing the CT-derived shape prior, our approach rectifies the inter-slice displacements and restores the continuity of structures, outperforming baseline methods in preserving anatomical fidelity.

#### 4.3.5. Analysis of the Surface-to-Volume Ratio

To evaluate the robustness of the proposed curvature intensity metric ($\bar{\kappa }$) against resolution variations, we calculated $\bar{\kappa }$ for cardiac substructures across five different spatial configurations, ranging from isotropic ($1\times 1\times 1\;{\mathrm{mm}}^{3}$) to anisotropic ($0.5\times 0.5\times 2\;{\mathrm{mm}}^{3}$) settings. As presented in Table 3, the metric demonstrates high stability; for instance, the LV values fluctuate only marginally between 0.113 and 0.129 despite changes in voxel aspect ratios. The standard deviation across resolutions is minimal compared to the inter-class differences between tissues (e.g., Myocardium vs. Atria). This stability confirms that $\bar{\kappa }$ primarily reflects the inherent topological complexity of the anatomical structure rather than being an artifact of the discretization grid.

**Figure 7:**
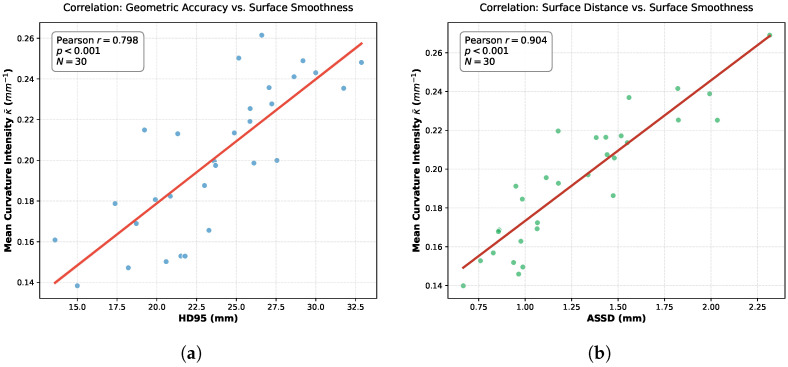
The correlation between the proposed surface-to-volume ratio metric and standard surface smoothness metrics. (**a**) Correlation between $\bar{\kappa }$ and HD95. (**b**) Correlation between $\bar{\kappa }$ and ASSD.

To further validate the clinical relevance of the surface-to-volume ratio, we analyzed its relationship with standard surface distance metrics using the validation cohort. Figure 7 displays the scatter plots correlating $\bar{\kappa }$ with the HD95 and the ASSD. We observed strong positive Pearson’s correlations of r=0.798 (p<0.001) for HD95 and r=0.904 (p<0.001) for ASSD. This strong linear relationship indicates that the surface-to-volume ratio serves as an effective proxy for geometric fidelity; an increase in $\bar{\kappa }$, indicating a higher surface area relative to volume, reliably corresponds to the presence of geometric outliers and surface irregularities typical of motion artifacts.

#### 4.3.6. Robustness of Noisy Labels

**Figure 8:**
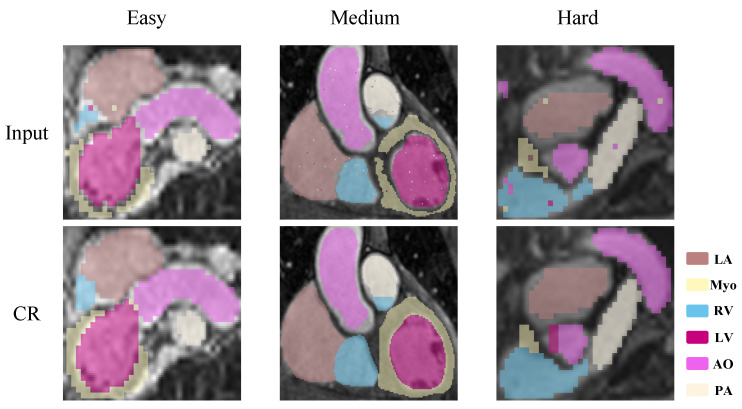
Visualization of robustness results for noisy labels.

To evaluate the robustness of our method against segmentation errors, we conducted an experiment using input labels corrupted with varying levels of synthetic noise. We simulated segmentation defects by applying random noise and boundary perturbations to the input MRI labels before feeding them into the network. As shown in Figure 8, when boundary perturbations and scattered label noise are introduced across different difficulty cases, the corrected results exhibit smoother ventricular contours and improved inter-slice continuity. Even under severe cases, the model suppresses fragmented regions and restores anatomically coherent chamber structures. This demonstrates that the CT-derived shape prior provides effective structural regularization against segmentation noise while preserving anatomical plausibility.

### 4.4. Super-Resolution Study

#### 4.4.1. Effectiveness of SR

To validate the generative capability of the ASV module, we conducted qualitative evaluations on the real MRI test set. Prior to super-resolution, all input volumes underwent motion correction via the DSL stage. This ensures that the inputs are spatially aligned, allowing the super-resolution network to focus on recovering through-plane details from the corrected low-resolution data rather than compensating for geometric displacements.

**Figure 9:**
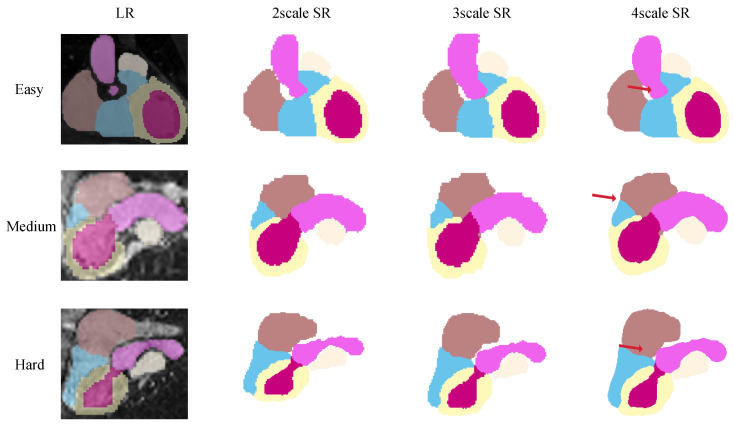
The 2D visualization of super-resolution results at different motion correction levels (easy, medium, hard). The proposed method consistently restores anatomically coherent ventricular structures across varying data quality.

**Figure 10:**
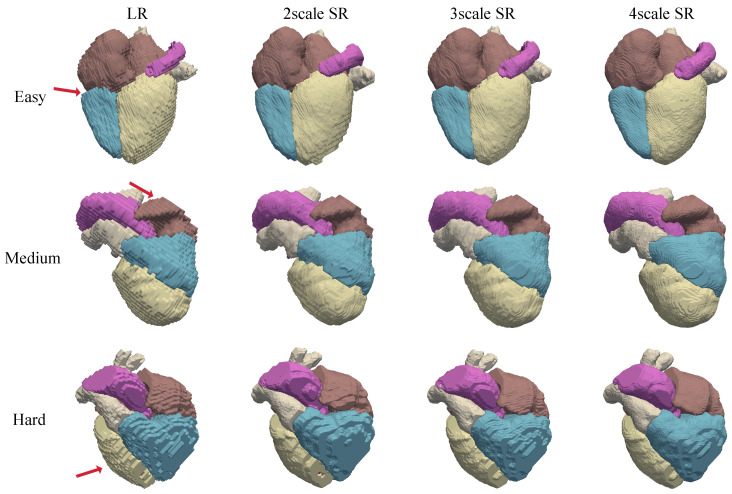
The 3D visualization of reconstructed super-resolved volumes, demonstrating improved anatomical continuity and fine structural fidelity compared to low-resolution baselines.

As shown in Figure 9, the proposed method produces anatomically coherent and topologically continuous reconstructions. From the 2D perspective, the corrected short-axis slices exhibit improved right ventricular morphology, where previously distorted contours are restored to anatomically plausible shapes. This correction leads to enhanced intra-slice structural consistency and smoother continuity across adjacent slices. From the 3D perspective (see Figure 10), the super-resolved segmentation volumes reveal richer structural details and sharper anatomical boundaries compared to their low-resolution counterparts. These results illustrate the ability of the model to synthesize high-fidelity volumetric labels that preserve both global cardiac geometry and fine-scale morphology.

#### 4.4.2. Effectiveness of SR Scales

To further examine the robustness of our method under different upsampling requirements, we conducted experiments on the real MRI dataset across multiple super-resolution scales (×2, ×3, and ×4). The input data were similarly pre-processed by the DSL module. To enable this multi-scale inference, the network was trained by dynamically downsampling the high-resolution CT ground truth by corresponding factors, allowing the INR to learn a resolution-independent continuous-shape manifold.

The results, as depicted in Figure 9 and Figure 10, indicate that the model maintains structural coherence and segmentation fidelity across all tested scales. Even at higher upsampling ratios (×4), where interpolation methods typically suffer from shape distortion or over smoothing, our approach reconstructs fine myocardial boundaries and cavity interfaces with consistent topology. This demonstrates the scalability of the ASV framework, which can flexibly adapt to the diverse spatial resolution requirements encountered in clinical cardiac MRI protocols.

### 4.5. Relationship Study of Motion Correction on Super-Resolution

#### 4.5.1. Visual Analysis on Clinical MRI

To explore the necessity of motion correction for super-resolution tasks in a clinical context, we conducted comparative experiments on the real MRI dataset. Specifically, we generated reconstructions based on two experimental conditions: one where the input data were processed directly by the SR module without prior correction, and another where the data were first aligned using the DSL motion correction framework. This comparison aims to visually verify whether geometric alignment is a prerequisite for effective volumetric upsampling.

**Figure 11:**
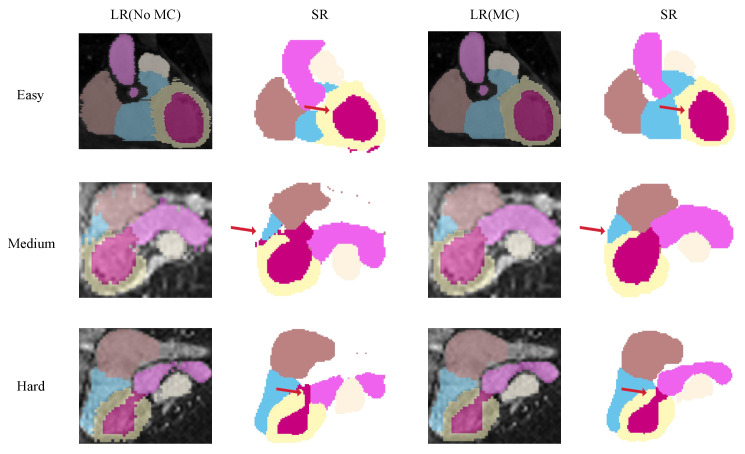
Comparative visualization of super-resolution results on MRI data with and without prior motion correction. The visual evidence demonstrates that applying super-resolution to uncorrected data amplifies inter-slice artifacts, whereas pre-aligning the data effectively restores high-granularity anatomical details and superior structural fidelity.

We examine the influence of motion correction on super-resolution performance by comparing reconstructions generated with and without the alignment module. As shown in Figure 11, performing super-resolution directly on misaligned inputs inevitably exacerbates spatial discontinuities, yielding anatomically implausible structures. In contrast, prior motion correction effectively restores slice-to-slice correspondence, allowing the super-resolution network to focus on recovering fine-grained details rather than struggling with geometric inconsistencies. The benefits are most pronounced in the right ventricle, where the motion-corrected reconstructions exhibit significantly enhanced global shape accuracy and volumetric coherence compared to their uncorrected counterparts. This visual evidence confirms that motion correction is a critical prerequisite for generating structurally faithful, high-resolution cardiac labels.

#### 4.5.2. Ablation Study on Simulated MRI

To quantitatively investigate the impact of motion correction strategies on super-resolution performance, we conducted an ablation study on the Simulated MRI dataset. Using the available ground truth, we calculated Dice, HD95, and ASSD metrics to objectively analyze how different alignment pre-processing steps ranging from no correction to our proposed method, affect the final super-resolved output.

As shown in Table 4, we compared four experimental settings: direct super-resolution without correction (w/o MC), and super-resolution combined with rigid registration, non-rigid registration, and our proposed latent space correction.

Impact of motion correction on SR: The baseline method without motion correction yielded the lowest performance across all metrics (Dice: 78.72%, HD95: 6.58 mm). This result indicates that direct super-resolution is insufficient when handling data with severe inter-slice misalignment. Since SR algorithms typically rely on local spatial continuity to enhance resolution, the presence of slice displacement introduces ambiguity, causing the network to generate artifacts rather than recovering true anatomical details. This confirms that effective motion correction is a necessary prerequisite for high-quality 3D volume reconstruction.

Comparison of geometric registration strategies: Implementing explicit registration methods improved the segmentation accuracy. Rigid registration, which aligns slices based on global translation and rotation, increased the Dice score to 82.15%. However, its effectiveness is limited as it cannot address the non-linear local deformations caused by soft tissue motion. Non-rigid registration further improved performance (Dice: 85.04%, HD95: 5.41 mm) by allowing for local pixel-wise warping. Despite this improvement, traditional non-rigid methods operate mainly on image gradients or intensity similarity. Without a semantic understanding of cardiac anatomy, they may produce geometrically smooth but anatomically inaccurate shapes, particularly in regions with large slice gaps.

Effectiveness of latent space mapping (ours): The proposed method achieved the best performance, with a Dice score of 88.36% and the lowest HD95 of 4.62 mm. Unlike explicit registration methods that warp the image in the spatial domain, our approach maps the misaligned volume into a compact latent volume map. By projecting the input data onto a learned anatomical manifold derived from high-quality priors, the network implicitly reconstructs a 3D volume that is both structurally consistent and topologically valid. This demonstrates that for the task of super-resolution, leveraging a learned structural prior is more effective than geometric deformation in resolving complex inter-slice inconsistencies.

#### 4.5.3. Analyze the Size of the Finetuning Dataset

To evaluate the trade-off between annotation effort and performance gain, we conducted an ablation study by varying the number of manually corrected MRI volumes used during the finetuning stage. As detailed in Table 5, the size 0 condition represents a direct application of the CT-pretrained model to MRI data, and it achieves a baseline Dice of 0.8465, demonstrating the robustness capability of the learned shape manifold. Notably, introducing a small set of target domain data substantially enhances precision. finetuning with just 7 volumes improves the Dice score to 0.8683 and reduces the HD95 by over 2 mm. Increasing the dataset size to 15 further refines the results to a Dice of 0.8778 and an HD95 of 22.88 mm. These results suggest that while more data yields better performance, our framework is highly data-efficient, achieving competitive reconstruction quality with a minimal annotation burden, thus maintaining its practical viability for clinical deployment.

#### 4.5.4. Computational Efficiency Analysis

To assess the clinical feasibility of DeepWHR, we evaluated the computational efficiency of our method against established baselines. Table 6 details the average inference time per case and the floating-point operations (FLOPs) required for reconstruction. Despite incorporating an implicit neural representation module, which is traditionally computationally intensive, our method achieves the lowest inference latency (1.3840 s) and computational cost (992.96 G FLOPs). This efficiency stems from our architecture’s design, which leverages a compact latent volume map to guide the coordinate-based queries, avoiding the computational burden of iterative optimization or dense voxel-wise deformation field generation found in competing approaches.

## 5. Discussion

In this study, we validated DeepWHR against state-of-the-art reconstruction (including motion correction and super-resolution) methods. Quantitatively, our framework achieved higher accuracy, outperforming competing approaches with the highest Dice score of 0.8778 and reduced boundary errors on simulated datasets. Qualitatively, unlike intensity-based or rigid correction methods that often retained stair-step artifacts or generated unnatural deformations, DeepWHR consistently restored smooth, anatomically coherent structures. This performance advantage is derived from our unique cross-modality mechanism, which learns the inherent isotropy and integrity of CT data. By leveraging high-fidelity CT prior to define a valid latent shape manifold, our network rectifies geometric inconsistencies before employing implicit neural representations, ensuring both topological validity and high-resolution morphological fidelity even under motion corruption.

In clinical practice, input labels can be readily obtained using state-of-the-art segmentation networks, such as nnU-Net or large-scale medical foundation models. Our framework then serves as a post-processing refinement step to correct motion artifacts and enhance resolution. Regarding segmentation errors, our method demonstrates resilience to small-scale inaccuracies. As the model learns a shape prior from high-resolution CT data, it can effectively smooth out jagged boundaries and correct minor topological defects caused by segmentation noise. However, large-scale segmentation failures may negatively affect performance. The framework is robust to moderate noisy labels, but it cannot recover a completely missing anatomical structure if its label is absent. Furthermore, the current pipeline is label-driven, and direct MRI intensity reconstruction is beyond its scope.

To simulate MRI artifacts and slice misalignment, we employed a degradation strategy on CT data. We simulated MRI’s low through-plane resolution via anisotropic downsampling and modeled inter-slice misalignment through random slice-wise translation and rotation. Additionally, random noise was injected to mimic motion-induced fuzziness. Although clinical MRI suffers from radiometric artifacts like bias fields and motion artifacts like respiratory motion, these primarily result in initial segmentation errors. Our framework addresses this via label-domain structural restoration. By learning a robust anatomical shape prior from CT data, the network can effectively rectify the discontinuous and implausible segmentation masks produced from the original MRI scans. The visualization results on real clinical MRI datasets confirm that our method successfully transforms sparse, artifact-ridden labels into smooth, high-resolution 3D models.

Despite these advancements, the current framework presents certain limitations that merit further investigation. First, while our method significantly alleviates the dependency on large-scale MRI datasets with high quality, it does not entirely eliminate the need for target domain supervision. A small subset of manually corrected MRI data is still required during the fine tuning stage. This step is critical to prevent the model from overfitting to CT morphology and ensuring that the inference results do not become overly biased toward CT-specific features. Second, regarding the flexibility of the super-resolution module, although the implicit representation supports continuous sampling, the current optimization strategy is not fully resolution agnostic. To achieve optimal performance across widely varying upsampling factors (e.g., shifting from ×2 to ×8), the model typically requires retraining or specific data augmentation adjustments. Developing a unified architecture that supports arbitrary-scale inference without retraining remains an area for future improvement.

Looking forward, the generation of labels free from motion artifacts and with high spatial resolution opens vast possibilities for advanced computational cardiology. We plan to incorporate more complex, non-rigid deformation models and physics-based artifact simulations in our future work to further improve the robustness of the framework. Furthermore, in the domain of computational fluid dynamics (CFD), the restored smooth boundaries of the ventricular cavity and aorta can effectively prevent artificial turbulence calculation caused by jagged surfaces, thereby improving the reliability of hemodynamic parameters such as wall shear stress. Another promising direction is the integration of these anatomically consistent labels into electrophysiological simulation pipelines, where topological defects in mesh generation often lead to erroneous conduction blocks. Ultimately, we aim to bridge the gap between routine clinical imaging and advanced physiological modeling, providing a reliable geometric foundation for the diagnosis and treatment planning of cardiovascular diseases.

## 6. Conclusions

This study presents a unified framework for reconstructing cardiac segmentation labels from MRI data corrupted by motion. By integrating DSL with ASV, our approach shifts the restoration paradigm from intensity-based alignment to structural rectification. We leverage anatomical priors derived from CT to address inter-slice misalignment and restore 3D structural integrity. The sequential transfer learning strategy effectively balances modality-invariant topological constraints with MRI-specific morphological characteristics, ensuring that the reconstructed labels inherit the geometric robustness of CT while preserving the intrinsic spatial distribution of the patient-specific anatomy. Future work will focus on incorporating non-rigid deformation models and physics-based simulations to further enhance robustness while extending the framework to support advanced computational cardiology tasks, such as high-fidelity computational fluid dynamics and electrophysiological modeling for reliable clinical applications.

## Figures and Tables

**Table 1:** Cardiac chamber volumes (mean ± standard deviation) in cm^3^.

Chamber	LV	RV	LA	RA	Myo	AO	PA
Volume (cm^3^)	59.8 ± 30.7	74.8 ± 21.9	92.1 ± 20.2	88.5 ± 23.6	114.4 ± 34.3	50.8 ± 16.4	51.0 ± 16.3

**Table 2:** Performance comparison of our method with other methods on Simulated MRI datasets. Best results are highlighted in bold.

Simulated MRI			
**Method**	**Dice (↑)**	**HD95 (mm ↓)**	**ASSD (mm ↓)**
Intensity [11]	0.8620	26.2063	1.6486
RCH3 [31]	0.8057	47.6205	23.0283
ACNNs [12]	0.8492	33.4788	1.9044
Chen [14]	0.8659	25.9304	1.3831
Wang [3]	0.8390	45.4953	2.0229
Ours	**0.8778**	**22.8842**	**1.3429**

**Table 3:** The mean curvature intensity ($\bar{\kappa }$) (surface-to-volume ratio, unit: ${\mathrm{mm}}^{-1}$) under different spacing configurations for cardiac substructures.

Spacing (${\mathrm{mm}}^{3}$)	Myo	LA	LV	RA	RV	AO	PA
1×1×1	0.243	0.112	0.116	0.114	0.135	0.169	0.164
1×1×2	0.240	0.116	0.114	0.116	0.139	0.167	0.166
1×1×4	0.249	0.117	0.113	0.118	0.138	0.160	0.167
0.5×0.5×1	0.255	0.122	0.129	0.129	0.146	0.179	0.175
0.5×0.5×2	0.252	0.120	0.126	0.127	0.141	0.176	0.172

**Table 4:** Ablation study on simulated dataset with different motion correction strategies. Best results are highlighted in bold.

Method	Dice (%) ↑	HD95 (mm) ↓	ASSD (mm) ↓
w/o MC	78.72	6.58	2.31
Rigid + SR	82.15	5.72	2.05
Non-rigid + SR	85.04	5.41	1.92
Ours	**88.36**	**4.62**	**1.68**

**Table 5:** Ablation study on the sensitivity of model performance to the size of the finetuning dataset on Simulated MRI datasets. Best results are highlighted in bold.

Simulated MRI			
**Size**	**Dice (↑)**	**HD95 (mm ↓)**	**ASSD (mm ↓)**
0	0.8465	26.2823	1.5102
7	0.8683	24.1786	1.3764
15 (Ours)	**0.8778**	**22.8842**	**1.3429**

**Table 6:** Comparison of average inference time and computational cost (FLOPs) across methods. Best results are highlighted in bold.

Method	RCH3	ACNNs	Chen	Wang	Ours
Average inference time (sec/per case)	1.6832	1.6593	1.7343	1.7544	**1.3840**
Average FLOPs (G)	1023.92	1138.34	1293.43	1283.56	**992.96**

## Data Availability

The original data presented in this study are openly available in CARE2024 WHS Challenge at https://zmic.org.cn/care_2024/track5/ (accessed on 24 December 2024). The code for this research is publicly available at https://github.com/Dongjinweicn/DeepWHR (accessed on 18 February 2026).
